# The Transcriptome of *Schistosoma mansoni* Developing Eggs Reveals Key Mediators in Pathogenesis and Life Cycle Propagation

**DOI:** 10.3389/fitd.2021.713123

**Published:** 2021-08-18

**Authors:** Zhigang Lu, Geetha Sankaranarayanan, Kate A. Rawlinson, Victoria Offord, Paul J. Brindley, Matthew Berriman, Gabriel Rinaldi

**Affiliations:** 1 Wellcome Sanger Institute, Wellcome Genome Campus, Hinxton, United Kingdom; 2 Department of Microbiology, Immunology & Tropical Medicine, and Research Center for Neglected Diseases of Poverty, School of Medicine and Health Sciences, The George Washington University, Washington, DC, United States

**Keywords:** Schistosoma mansoni, eggs, RNAseq, early embryogenesis, late embryogenesis, neglected tropical disease (NTD)

## Abstract

Schistosomiasis, the most important helminthic disease of humanity, is caused by infection with parasitic flatworms of the genus *Schistosoma*. The disease is driven by parasite eggs becoming trapped in host tissues, followed by inflammation and granuloma formation. Despite abundant transcriptome data for most developmental stages of the three main human-infective schistosome species—*Schistosoma mansoni, S. japonicum* and S. *haematobium*—the transcriptomic profiles of developing eggs remain under unexplored. In this study, we performed RNAseq of *S. mansoni* eggs laid *in vitro* during early and late embryogenesis, days 1-3 and 3-6 post-oviposition, respectively. Analysis of the transcriptomes identified hundreds of up-regulated genes during the later stage, including venom allergen-like (VAL) proteins, well-established host immunomodulators, and genes involved in organogenesis of the miracidium larva. In addition, the transcriptomes of the *in vitro* laid eggs were compared with existing publicly available RNA-seq datasets from *S. mansoni* eggs collected from the livers of rodent hosts. Analysis of enriched GO terms and pathway annotations revealed cell division and protein synthesis processes associated with early embryogenesis, whereas cellular metabolic processes, microtubule-based movement, and microtubule cytoskeleton organization were enriched in the later developmental time point. This is the first transcriptomic analysis of S. *mansoni* embryonic development, and will facilitate our understanding of infection pathogenesis, miracidial development and life cycle progression of schistosomes.

## Introduction

Schistosomes are parasitic flatworms that infect more than 250 million people worldwide, mainly in Low and Middle-Income Countries (1). There is only a single effective drug (praziquantel), and an ongoing threat of drug resistance emerging (2). While adult worms can dwell within the blood vessels of humans for years, it is their eggs rather than the worms themselves that drive pathology (3). It is estimated that one pair of *Schistosoma mansoni* adult worms can lay >300 eggs per day (4). Once the eggs are laid by the worms in the mammalian bloodstream, about half migrate, over the course of six days, through the endothelium of blood vessels, across the epithelium of the gut and are released into the intestinal lumen (5–7). Eggs that reach water hatch into miracidium larvae that must infect a freshwater snail to continue the life cycle. The remaining eggs are swept around the body by the bloodstream and become trapped in host tissues, mainly in the liver, intestines and spleen, where they induce immune responses, severe inflammation and granuloma formation (8). The granuloma surrounding the trapped egg consists of an immune cellular complex that includes macrophages, lymphocytes and eosinophils (3). In addition, fibroblasts in the granuloma produce collagen that leads to periportal fibrosis and induction of collateral circulation, including varices, which increase the risk of life-threatening haemorrhage in the digestive tract (9).

The study of the schistosome egg and its interaction with the host is critical to understand not only the pathogenesis associated with the infection, but also parasite strategies to exit the mammalian host to perpetuate the life cycle (6,7). Thus, numerous reports have focused on soluble egg antigens (SEA) and several excreted-secreted products, including proteins and glycans that interact with the host tissues, inducing immune responses and facilitating the egress of the egg to the external environment (10–14). Notably, the immune modulatory roles of egg-specific antigens, such as Omega, Kappa and IPSE have been validated by functional approaches such as shRNA-mediated knock-down (15) and CRISPR-Cas-based genome editing (16). More recently, the molecular and cellular mechanisms involved in granuloma formation have been dissected using a zebrafish model of macrophage dependent granuloma induction (17). This novel infection model demonstrated that host and parasite molecules play key roles in shaping the granulomatous response and that the response is dependent on the level of egg maturity (17,18).

Notwithstanding this progress, few studies have focused on the development of the miracidium within the egg capsule (19). Jurberg et al. (20) provided a detailed morphological description of embryogenesis of the miracidium inside the egg capsule while migrating through the host tissue. In addition, these investigators provided a revised staging scheme for the miracidial development that comprises eight discrete stages (20). No transcriptome analysis underlying this developmental progression has yet been performed, with only a single RNA-seq report of *S*. *mansoni* eggs isolated from the liver of experimentally-infected hamsters (21). Aiming to address this information deficit, we performed comparative transcriptomics (RNA-seq) on *in vitro* laid eggs of *S*. *mansoni* at two time points - early and late embryogenesis. More than 1,300 genes were differentially expressed, including up-regulated in the late development stage of genes associated with organogenesis of the miracidium. The investigation revealed transcriptional signatures in developing *in vitro* laid eggs that will facilitate our understanding of the pathogenesis associated with the infection, miracidial development and life cycle progression of schistosomes.

## Material and Methods

### Ethics Statement

The complete life cycle of the NMRI (Puerto Rican) strain of *S. mansoni* is maintained at the Wellcome Sanger Institute (WSI) by breeding and infecting susceptible *Biomphalaria glabrata* snails and mice. The mouse experimental infections and other regulated procedures were conducted under the Home Office Project Licence No. P77E8A062 held by GR. All protocols were revised and approved by the Animal Welfare and Ethical Review Body (AWERB) of the WSI. The AWERB is constituted as required by the UK Animals (Scientific Procedures) Act 1986 Amendment Regulations 2012.

### *In Vitro* Laid Eggs

Schistosome eggs laid *in vitro* by cultured adult worms (*in vitro* laid eggs or IVLE) were collected as described (22). Briefly, mixed-sex adult worms were recovered from mice by portal perfusion 6 weeks after infection, washed in 1X PBS supplemented with 200 U/ml penicillin, 200 mg/ml streptomycin and 500 ng/ml amphotericin B (ThermoFisher Scientific), transferred to 6-well plates, and maintained in culture in complete Basch media at 37°C in 5% CO_2_ (23). All media components were purchased from ThermoFisher Scientific. The eggs laid by the worms in culture during the first 72 h post-perfusion were recovered. Fifty percent of the eggs were collected at this time for the early embryogenesis samples (D3 eggs), concentrated by gravity, resuspended in 750 μl Trizol reagent, snap frozen and stored at -80°C. The remaining IVLE were cultured for a further 3 days to allow further development before collection for the late embryogenesis samples (D6 eggs) (24). A total number of eggs ranging from 500 to 1000 IVLE were collected at each time point. We performed a separate collection of IVLE, as above, for each of three independent perfusions of adult schistosomes from experimentally infected mice.

### RNA Extraction From *In Vitro* Laid Eggs

IVLE frozen in Trizol reagent were subjected to 3 freeze-thaw cycles by manually transferring tubes between a water bath at 95°C and dry ice. This procedure enhanced the total RNA yield from the samples. Thereafter, the eggs were transferred to MagNA Lyser tubes (Roche) containing ceramic beads, homogenized in FastPrep (FastPrep-24, MP Biomedicals) at setting 6 with two 20-second pulses and incubated for 5 minutes at room temperature. To each sample, 150μl chloroform was added, shaken vigorously for 10 seconds, incubated for 3 minutes at room temperature and centrifuged at 15,000g for 15 minutes at room temperature. The aqueous phase was carefully removed to a clean centrifuge tube, and the RNA was precipitated by adding one volume of 100% ethanol and incubating at -80°C overnight. The samples were centrifuged at maximum speed for 30 minutes at 4°C, the RNA pellet washed in 70% ethanol, air dried and resuspended in nuclease free water. The RNA quality was assessed and quantified using the Bioanalyzer (2100 Bioanalyzer Instrument, Agilent Technologies). Although the yield of RNA was modest, <30 ng total, high-quality RNA was recovered from each replicate sample (Supplementary Figure S1A).

### Library Preparation and Sequencing

Given the minimal amount of RNA obtained from IVLE, the Smart-Seq2 protocol was adapted for low-input RNA library preparation (25). Two different amounts of input RNA, 12 ng and 3 ng, for each sample and its replicates were prepared. Polyadenylated mRNA was enriched using 5 mg/ml Dynabead Oligo(dT)_20_ in 1X PBS (pH 7.4) from mRNA DIRECT kit (Thermo Fisher). Beads were washed with 10mM Tris-HCl pH 7.5, 150 mM LiCl, 1mM EDTA pH 8.0, 0.1% w/v LiDS in nuclease free water and RNA eluted using 10 mM Tris-HCl pH 8.0 at 75°C for 2 minutes. The poly A-enriched RNA was reverse transcribed by Smart-Seq2 as described (25) with 10 reverse transcriptase cycles, and cDNA further amplified using IsoSeq PCR (ISPCR) primers with 10 or 11 PCR cycles. Dual indexed sequencing libraries were made out of 5 ng cDNA from the above preparations using Illumina Nextera library preparation kit according to manufacturer’s instructions (Illumina). Quality checked and equimolar pooled libraries were sequenced in a HiSeq 4000 Illumina system and 75bp paired- non-stranded- reads generated. Sequence data were deposited in the European Nucleotide Archive (ENA) with the study number ERP128933; NCBI BioProject ID PRJEB44842 (accession numbers for each sample are shown in Supplementary Table S1).

### Mapping of RNA-Seq Reads and Gene Counting

Sequence reads from eggs isolated from the liver of experimentally-infected hamsters 6 to 8 weeks after infection (‘liver eggs’) were obtained from published data (21). The reads were mapped to *S*. *mansoni* v7 genome (WormBase Parasite WBPS14) using Hisat 2.1.0 (26) due to unequal read lengths generated on Roche 454. Sequence reads for IVLE were mapped using STAR 2.5.0a (27) with the option –alignIntronMin 10. Counts per gene were summarised with FeatureCounts v1.4.5-p1 (28) based on the exon feature, using the annotation from WormBase Parasite WBPS14 (https://parasite.wormbase.org/) (29).

### Differential Gene Expression Analysis

Raw read counts from both liver eggs (21) and IVLE samples (this study) were combined and used as input for DESeq2 v1.26.0 (30). The Pearsons’s correlations between replicates were examined. For differential expression analysis, we set cooksCutoff=TRUE to remove extreme outlier genes. Genes with adjusted **p-**value (Padj) < 0.01 and fold-difference > 2 were regarded as significantly differentially expressed. Log-transformed count data were used for principle component analysis (PCA) and calculating the Euclidean distance between samples.

### Gene Ontology Enrichment Analysis of Differentially Expressed Genes

Gene Ontology (GO) annotation for *S*. *mansoni* genes were obtained by running InterProScan v5.25 (31). Enrichment analysis of differentially expressed genes (DEGs) was performed using topGO v2.38.1 (32), with 5 nodes and the weight 01 method. GO terms with FDR < 0.05 were considered as significantly enriched. Gene product descriptions were obtained using the Biomart tool at WormBase Parasite (https://parasite.wormbase.org/).

### Enrichment of Pfam Family and InterPro Domains

The annotations of Pfam family and InterPro domain in *S. mansoni* gene products were obtained from InterProScan v5.25. Analysis of functional enrichment in DEGs was conducted *via* Fisher’s Exact test followed by a *p*-value correction using the Benjamini-Hochberg procedure. Terms with FDR < 0.05 were considered as significant.

### KEGG Pathway Mapping

Mapping of *S. mansoni* gene products to the KEGG pathway database was performed on the KAAS server (https://www.genome.jp/kegg/kaas/) using the GHOSTX program and BBH method. The significance of DEGs enrichment in pathways was assessed using Fisher’s Exact test and resulting *p*-values were adjusted using the Benjamini-Hochberg procedure for each of KEGG categories 1-5 (https://www.genome.jp/kegg/pathway.html; excluding pathways for prokaryotes, yeast, and plant). Pathways with FDR < 0.05 were considered as significant. The scripts used for functional enrichment (GO, Pfam, InterPro, KEGG) analysis can be accessed at https://github.com/zglu/Gene-function-enrichment.

## Results

### Transcriptional Signatures Underlie the *In Vitro* Development of *Schistosoma mansoni* Eggs

**Figure 1:**
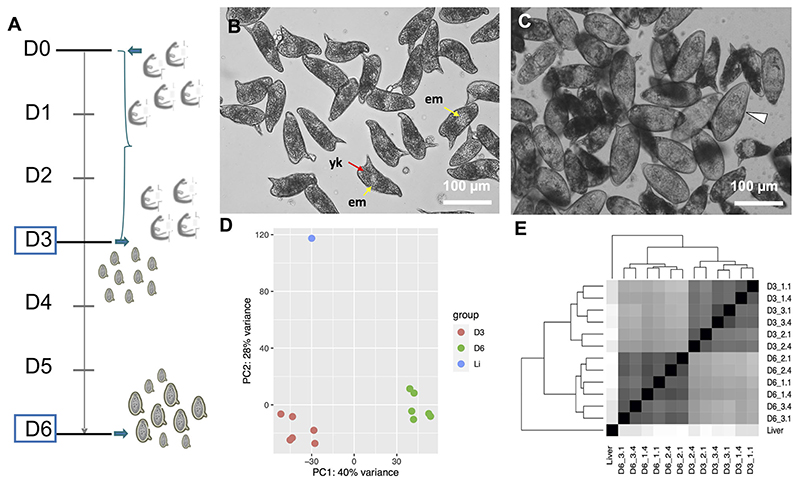
**(A)** Timeline depicting the experimental design. On day 0 (D0) adult worms perfused from infected mice were washed and placed in culture for three days. The worms laid eggs, i.e. *in vitro* laid eggs (IVLE), for three days (bracket). On day 3 (D3) the worms were removed from the culture, and half of the IVLE were collected for RNAseq - D3, early embryogenesis sample. The remaining IVLE were cultured for three more days, and on day 6 (D6) they were collected for RNAseq - D6, late embryogenesis sample. **(B, C)** Representative micrographs of 3 days old- **(B)** and 6 days old- **(C)** *in vitro* laid eggs (IVLE). Scale bar: 100 μm. *em*, embryo (yellow arrow); *yk*, yolk (red arrow); white arrowhead, fully developed egg containing the mature miracidium. **(D)** Clustering of egg samples using Principal Component Analysis. D3, D3 IVLE; D6, D6 IVLE; Li, liver eggs **(E)** Clustering of egg samples using Sample Distance Matrix. Names of samples are described in Supplementary Table S1.

To study the transcriptional profiles associated with *in vitro* development of eggs, we performed RNA-seq on samples corresponding to early embryogenesis, 1–3 days post-oviposition (D3), and late embryogenesis, 4–6 days post-oviposition (D6) (Figure 1A). According to previously described criteria, most D3 eggs belonged to stages I (nonvisible embryo under the light microscope) and II (visible embryo as a clear central disk that occupies one third of the egg) (19, 20) (Figure 1B and Supplementary Figure S2A). By D6, the eggs were further developed and had increased in size by one third, as previously reported (20). In addition, 40–45% of the D6 eggs had progressed to stages III (enlarged embryo that occupies two thirds of the egg length), IV (embryo occupying almost the entire egg), or V (fully mature miracidium inside the eggshell before hatching, some motile miracidia) (19, 20) (Figure 1C and Supplementary Figure S2B and Supplementary Video S1).

The quality of the total RNA extracted from IVLE was much higher than that of the highly-degraded RNA usually obtained from eggs collected from the liver of experimentally-infected mice (Supplementary Figure S1B). On the other hand, the total RNA yield isolated from ~500–1000 IVLE was <30 ng. Therefore, we adapted a Smart-Seq2 protocol originally designed for single-cell RNA-seq (25) to produce high-quality RNA-seq libraries from 3 or 12 ng of input RNA. We obtained 0.2–3.3 million RNA-seq raw reads per library, with three-quarters of them having at least two-fold more reads than the published liver egg sample (21) (0.36 million reads; Supplementary Table S1). We note that the latter sample was sequenced using Roche 454 sequencing technology and 1000 ng of polyA^+^ RNA. All replicates of IVLEs showed good correlations (Pearson’s *r* > 0.83; Supplementary Table S2) and similar numbers of detected genes (counts per million, CPM > 10; Supplementary Table S1). From both Principal Component Analysis (PCA) and Sample Distance Matrix analyses, the samples clustered according to their developmental stage (Figures 1D, E).

### *In Vitro* Developed Eggs Are Transcriptionally Distinct From Eggs Collected From the Host

**Figure 2:**
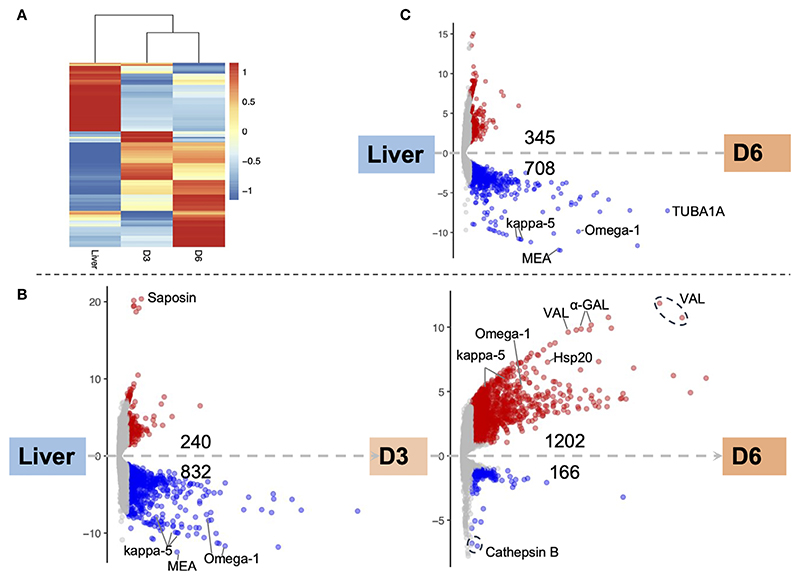
Differential gene expression among egg samples. **(A)** Hierarchical clustering showing divergent transcriptomic signatures among the three samples. Liver, liver eggs; D3, D3 IVLE; D6, D6 IVLE. The colour scale indicates the Z-score values. **(B)** Volcano plots showing differentially expressed genes (DEGs) in D3 IVLE compared to liver eggs (left) and in D6 IVLE compared to D3 IVLE (right). Highlighted genes: Saposin (Smp_105420), kappa-5 (left: Smp_335470, Smp_335480 & Smp_335490; right: Smp_335490 & Smp_344300); Omega-1 (left: Smp_334170 & Smp_345790; right: Smp_345790), Hsp20 (Smp_302270), VAL (venom-allergen like protein - Smp_070250, Smp_176180 & Smp_120670), a-GAL (Alpha-N-acetylgalactosaminidase - Smp_247750 & Smp_247760), Cathepsin B (Smp_067060 & Smp_103610). **(C)** Volcano plot showing DEGs in D6 IVLE compared to liver eggs. Highlighted genes: kappa-5 (Smp_335470 & Smp_335480), MEA (major egg antigen - Smp_302350), Omega-1 (Smp_334170), TUBA1A (Tubulin alpha-1A chain - Smp_090120). In the volcano plots the *x-axes* represent -log10Padj values and the *y-axes* represent log2FoldChange. The volcano plots are available as interactive charts at http://schisto.xyz/IVLE/.

**Figure 3:**
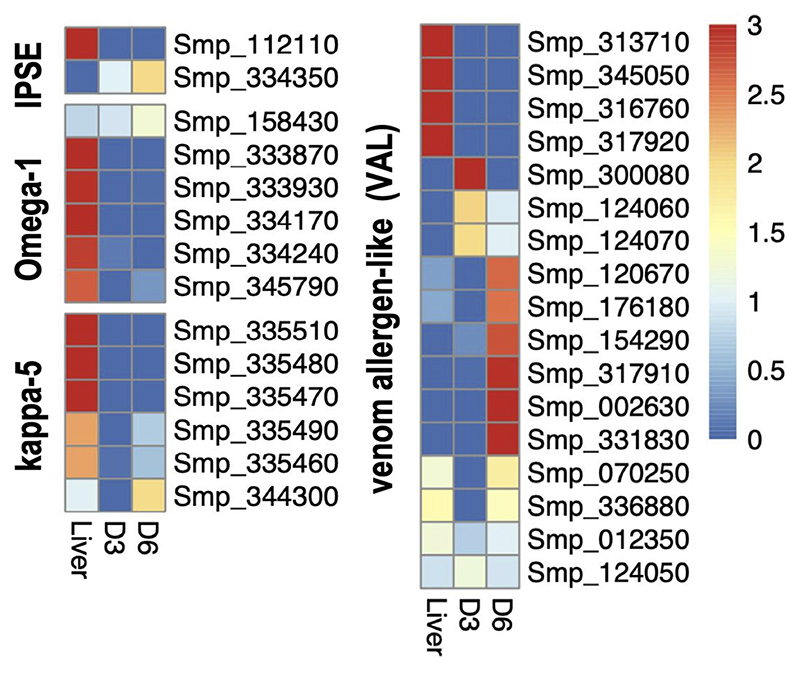
Heatmaps showing the relative gene expression of the egg antigens IPSE, Omega-1, kappa-5 and VALs in liver, D3 and D6 IVLE, as indicated. The colour scale shows relative values to the mean of each row using normalised counts from DESeq2.

Major transcriptomic differences were evident among the three egg samples: the two IVLE samples—early embryogenesis (D3), late embryogenesis (D6)—and the liver-collected eggs. However, the gene expression profiles of D3 and D6 IVLE partially overlapped suggesting progressive transcriptome changes during egg development (Figure 2A). Conversely, the transcriptome of liver eggs was distinct to that of IVLE; 240 and 832 genes, respectively, were significantly up- and down-regulated in D3 IVLE compared to liver eggs; 345 and 708 genes, respectively, were significantly up- and down-regulated in D6 IVLE compared to liver eggs (adjusted *p* < 0.01 & fold-difference >2; Figure 2 and Supplementary Figure S3, Supplementary Tables S3, S4). Strikingly, the expression of well-described immunomodulatory egg-specific genes, including *omega-1* (Smp_345790 & Smp_334170) and *kappa-5* (Smp_335470, Smp_335480 & Smp_335490) (15, 16) was significantly higher in liver eggs compared to IVLE (Figures 2B, C and 3, Supplementary Table S4). Meanwhile, one omega-1 (Smp_345790) and two kappa-5 (Smp_335490 & Smp_344300) genes showed more than a 30-fold increase in expression at D6 compared to D3 (Figure 2). In addition, expression of four the putative major egg antigens (Smp_302350, Smp_302340, Smp_247170 and Smp_302280), including one extensively studied as a key component in the T-cell-mediated response during the granuloma formation (33), significantly increased during *in vitro* egg development (Supplementary Table S4). In addition to these well-characterised immunomodulatory egg-specific genes, micro-exon genes (MEG) were also identified in D3 or D6 IVLE; however, only MEG 2 (Smp_159830) showed significant upregulation in D6 compared to D3 IVLEs (Supplementary Table S4).

### Miracidium-Enriched Genes Up-Regulated in Late Egg Development

Further pairwise analysis between both IVLE transcriptomes, showed 1202 up- and 166 down-regulated genes in D6 compared to D3 eggs (Figure 2B and Supplementary Table S4), indicating an overall upregulation of gene expression as embryogenesis proceeded. Of the 1202 upregulated genes in D6, 524 are among marker genes for different cell types previously defined by single cell RNA-seq in schistosomula, the first intra-mammalian stage (34). Of these, 53.8% (282/524) belong to neurons, 18.7% (98/524) to muscles, 18.9% (99/524) to the parenchyma tissue, and 8.6% (45/524) to germinal cells (Supplementary Table S5).

**Figure 4:**
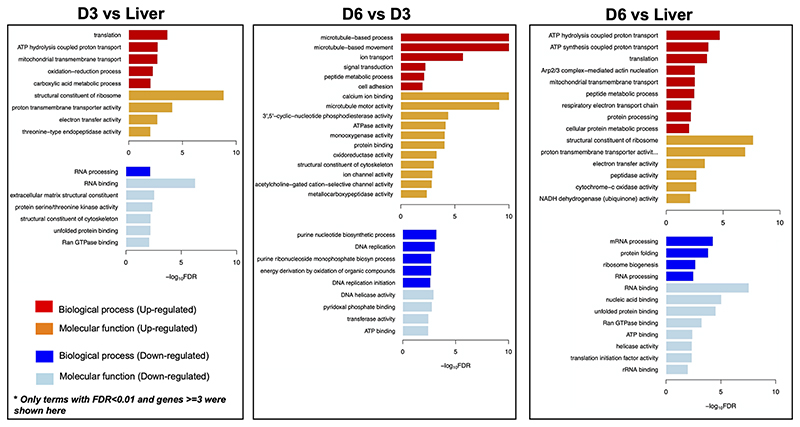
Gene Ontology (GO) enrichment in differentially expressed genes. For each comparison - D3 *vs* Liver; D6 *vs* D3; D6 *vs* Liver, only GO terms with FDR<0.01 and at least three genes are visualised. D3, D3 IVLE; D6, D^^^ IVLE; Liver, liver eggs.

Genes involved in the interaction between the miracidium and the snail were among the 10 with highest fold differences in expression between D6 and D3 eggs (Supplementary Table S4). For example, genes encoding venom allergen-like (VAL) proteins were upregulated in D6 eggs (Figure 3), including VAL 9 (Smp_176180), VAL 5 (Smp_120670), and VAL 15 (Smp_070250) (Figure 2B and Supplementary Table S4). Functional analysis based on Gene Ontology (GO) and KEGG Pathways revealed biological processes and molecular functions associated with differentially-expressed genes among the three egg samples (Figure 4 and Supplementary Table S6). The transcriptome of D3 compared to D6 eggs or liver eggs, showed an enrichment for DNA replication, cell cycle, ribosome biogenesis and RNA translation (Figure 4). This is consistent with the cell division and protein synthesis which are critical processes in the early developing embryo (20). In contrast, up-regulated genes in D6 eggs were associated with processes associated with movement and signalling, e.g. microtubule-based movement, signal transduction, and GPCR signalling pathways (FDR<0.05; Figure 4 and Supplementary Table S6). The microtubule motor activity associated with the later developmental time point may be related to the development of the ciliary plates of the miracidium, which enable swimming.

Several common features between the mature egg and the miracidium were identified, and indeed the presence of fully-developed miracidia within eggshells was evident in the D6 eggs (Figure 1B and Supplementary Figure S2B). Therefore, we asked whether a transcriptional footprint was found during the development of D6 eggs towards miracidium. To this end, we examined the top 100 genes that were previously shown to be enriched in miracidium-sporocyst (35) among all developmental stages, and found that around a third were significantly up-regulated in D6 eggs (Supplementary Table S7). The rest of the genes showed either no significant differential expression, or no expression in IVLEs (Supplementary Table S7). When considering the top 200 miracidium-enriched genes, only 4.4% were more abundant in D3 eggs, but 21.9% displayed higher expression in D6 eggs (Supplementary Tables S4,S7), including *Sm*VAL2 (Smp_002630) and *Sm*VAL15 (Smp_070250), which were previously shown to be highly expressed in the miracidium (36). In addition, in D6 eggs we found an evident upregulation of *tektin* (Smp_162540) and *tubulin* (Smp_079960) genes, which are essential for microtubule assembly and physiology, key components of the ciliary machinery.

## Discussion

The schistosome egg is the main driver of the chronic pathology associated with schistosomiasis (3). In addition, it is a developmental stage that ensures the propagation of the parasite from the definitive host to the intermediate host *via* the external environment. It has been recently shown that the timing of granuloma formation is actively manipulated by developing eggs, to avoid immune destruction or premature extrusion from the host (17). Thus, it is critical to understand the transcriptome landscape driving the egg development. Other than a handful of descriptive reports on embryogenesis (20) and egg secretions (37), the number of studies focused on gene expression changes in schistosome eggs seems surprisingly scarce. There is only one public RNAseq transcriptome dataset for *S*. *mansoni* eggs and one for *S*. *haematobium* eggs (21, 38). In addition, a few previous studies have employed expressed-sequence tags (ESTs) or microarrays to described changes in gene expression across developmental stages, including eggs of *S*. *mansoni* (39, 40) and *S*. *japonicum* (41, 42). Mass spectrometrically-determined proteomes of the soluble egg and secreted proteins of egg, recovered from liver of *S*. *haematobium-*infected mice, also have been reported (43). In all these studies, the eggs were isolated from livers of experimentally-infected rodents and the egg transcriptomes/proteomes were compared to those of male and female adult worms aiming at identifying genes involved in host-parasite interaction. The studies did not explore changes in gene expression during embryogenesis.

Investigation of the transcriptome of the schistosome egg has been impeded by intrinsic difficulties of this stage, including the presence of the eggshell and abundance of egg-derived RNases that degrade the transcripts during recovery of RNA. Here, we optimised a protocol to isolate high-quality RNA from eggs by consecutive rounds of freeze-thawing cycles. The quality of total RNA isolated from *in vitro* laid eggs (IVLE), even after 3 cycles of freeze-thawing was significantly superior to that of RNA isolated from liver eggs. Similarly, it has been reported that for biospecimens stored in biobanks, such as tumour sections, the RNA quality is optimal for downstream analyses after 3 freeze-thawing cycles, but it is dramatically negatively affected after five freeze-thaw cycles (44). In addition, we successfully employed the SmartSeq2 protocol (25) to produce bulk RNA-seq data starting with few nanograms of input RNA, as has been recently shown for transcriptomic studies in *Trichuris muris* larvae (45). Repurposing single-cell RNAseq protocols to generate high-quality bulk transcriptomic data from pico- to nanograms of total RNA or from few cells (46) becomes a promising approach when the amount of RNA is limited.

We have previously optimised the collection of schistosome IVLE, followed and quantified their daily development (24, 47). We have now produced RNA-seq data from developing eggs of *S*. *mansoni*. Although less total RNA was used for library preparation, we observed similar transcriptome coverage in IVLE compared to the only published data from liver eggs. Normalisation using the DESeq2 median-of-ratios method has also accounted for the differences in various library sizes, resulting in robust differential expression analysis (30). We observed drastic changes in the transcriptome profile of developing eggs and inferred functional roles of differentially expressed genes associated with embryo development (20). Our findings are consistent with descriptions of the egg development *in vitro* and the characterisation of excreted-secreted proteins by mature eggs (37). The authors describe highly abundant protein synthesis in 3-day cultured eggs compared to freshly isolated eggs. Our findings show that the up-regulated genes in D3 *vs* liver eggs and D6 *vs* D3 eggs are consistently associated with protein synthesis-associated processes such as translation, translational elongation, and regulation of phosphorylation. We found that genes with established immunomodulatory roles, e.g. *omega-1* (13), *kappa-5* (12) and the putative major egg antigen Sm-p40 (33) show a higher expression in mature eggs compared to immature eggs. However, the overall expression of these genes was much higher in eggs isolated from the host liver compared to IVLE suggesting that host factors may be required for driving their expression and/or stimulating the genesis of the subshell membrane where some of these proteins seem to be produced (12, 33). The eggs collected from the liver of experimentally-infected rodents likely comprised eggs ranging in developmental stage from newly laid to mature and likely also included eggs already dead due to immune responses and other factors. Thus, any comparison between the liver eggs and IVLE need to be taken cautiously. The *in vitro* culture conditions do not completely mimic the *in vivo* development of the schistosome and hence, the production of fully viable eggs is limited. This is consistent with the low percentage (ranging from 10% to 15%) of IVLE that hatch fully viable and infectious miracidia (24). Recent improvements in culture conditions offer novel and informative approaches to sustain and study *in vitro* and *ex vivo* parasite development, including sexual differentiation and fecundity (48–50).

Notwithstanding the limitations of our culture system, we identified a transcriptional footprint consistent with the developmental transition from D3 to D6 egg samples and from D6 egg samples towards the miracidium. In the D6 samples, we identified tissue-specific markers for muscle cells, nerve system, parenchymal and germ cells (34). We speculate that these genes may be involved in the specification and differentiation of diverse somatic and germinal tissues in the developing miracidia (51). Products of several genes upregulated in D6 eggs had previously been annotated as “larval transformation proteins” during *in vitro* miracidium-to-sporocyst transformation (52), including venom allergen-like proteins (*Sm*VALs) (36). The role of *Sm*VALs is not yet confirmed, but in some nematodes, VALs are involved in mechanisms of infection establishment and host immunomodulation (53). More recently, using a combination of RNAi and *in situ* hybridisation, Perally and colleagues demonstrated the critical role of *Sm*VAL6 in the maintenance of the tegumental barrier in adult worms (54). These findings suggest that the *Sm*VALs may be tentative targets for drug development. Whether *Sm*VALs, already upregulated in the mature egg, display similar functions during infection of the snail remains to be addressed. Proteomic approaches in *S. japonicum* identified proteins in the mature egg that are associated with miracidium motility (55). Similarly, we identified an upregulation of tektin (Smp_162540) and tubulin (Smp_079960) genes, both essential proteins for microtubule assembly and cilium physiology. These findings were consistent with previous microarray-based findings in which genes overexpressed in liver eggs were associated with microtubule motor activity, microtubule-based movement/process and cytoskeleton organisation and biogenesis (40). Remarkably, the authors also showed that the transcriptome profiles of the egg and daughter sporocyst are highly divergent, in contrast with those of egg and miracidium/mother sporocyst described in the current study. Here, a transcriptional landscape transition from D3–D6 egg samples to miracidium was revealed, with upregulation of previously described genes expressed in the mature egg such as the micro-exon gene 2 (MEG 2) (56). MEG may represent a novel mechanism for immune evasion by schistosomes (57).

In this study, we have reported the first transcriptome analysis of developing eggs from *S*. *mansoni*, central drivers of this major neglected tropical disease. Their transcriptomes have clear signatures of the parasite gearing up for life cycle progression, including key proteins required for the structure and motility of the miracidium and for the subsequent infection of snails. Along with highlighting proteins already known to drive egg-induced pathology, the transcriptome analysis has revealed dozens of other genes with similar profiles, not previously associated with pathogenesis but now warranting deeper investigation. Follow up studies on key genes involved in life cycle progression would reveal tentative targets for novel control strategies for this Neglected Tropical Disease.

## Supplementary Material

Figure S1 Total RNA isolated from different egg samples. **(A)** Bioanalyzer electropherogram of RNA preparations isolated from D3 and D6 IVLE and processed for sequencing using the Smart-Seq2 protocol. Samples and concentrations are indicated in the bottom panel. **(B)** Representative bioanalyzer traces of RNA preparations isolated from IVLE and liver eggs (LE), as indicated.

Figure S2 Representative micrographs of D3- **(A)** and D6 **(B)** IVLE. Scale bar: 300 μm.

Figure S3 **Left.** Venn diagram indicating the number of shared/unshared upregulated genes amongst the three comparisons: D6 *vs* D3 IVLE (1202 genes); D3 IVLE *vs* liver eggs (240 genes); D6 IVLE *vs* liver eggs (345 genes). **Right.** Venn diagram indicating the number of shared/unshared downregulated genes amongst the three comparisons: D6 *vs* D3 IVLE (166 genes); D3 IVLE *vs* liver eggs (832 genes); D6 IVLE *vs* liver eggs (708 genes). The gene names and identifiers are provided in **Supplementary Table S3**.

Figure S4 Pie chart indicating the differential expression of the top 200 miracidium-sporocyst enriched genes in D3 and D6 IVLE. Filtered: genes that were filtered out for differential expression analysis in DESeq2, as were detected as outlier genes; DEG: differentially expressed genes.

Supplementary video Representative video showing D6 IVLE. Fully mature, motile miracidia can be seen within their eggshells. Scale bar: 50 μm.

Table S1 RNA-Seq mapping statistics and accession numbers for all samples. Sample names refer to the day of the egg collection (D3 or D6), the number of the biological replicate (e.g. D3_1, D3_2, D3_3), and to the number of technical replicates employing 3 ng (1 μl - 1) or 12 ng (4 μl - 4), respectively.

Table S2 Pearson’s correlations among the biological replicates of the *in vitro* laid eggs.

Table S3 Gene IDs and normalised counts for the genes numbered in the Venn diagrams in **Supplementary Figure S3**.

Table S4 Lists of differentially expressed genes among liver eggs, D3, and D6 IVLEs.

Table S5 Lists of up-regulated genes in D6 (*vs* D3) IVLE that were enriched in different schistosomula cell clusters identified in (34).

Table S6 Enriched functions in identified differentially expressed genes (DEGs), includingGeneOntology, KEGGPathway, and Pfam/InterPro domains.

Table S7 List of differentially expressed genes in D3 and D6 IVLEs enriched in miracidium-sporocyst.

## Data Availability

The datasets presented in this study can be found in online repositories. The names of the repository/repositories and accession number(s) can be found in the article/ Supplementary Material.
