# Supplementary figures and images for: The Transcriptome of Schistosoma mansoni Developing Eggs Reveals Key Mediators in Pathogenesis and Life Cycle Propagation
Source: Front Trop Dis. Author manuscript; Available in PMC 2022 Nov 15. (PMC7613829; doi:10.3389/fitd.2021.713123)

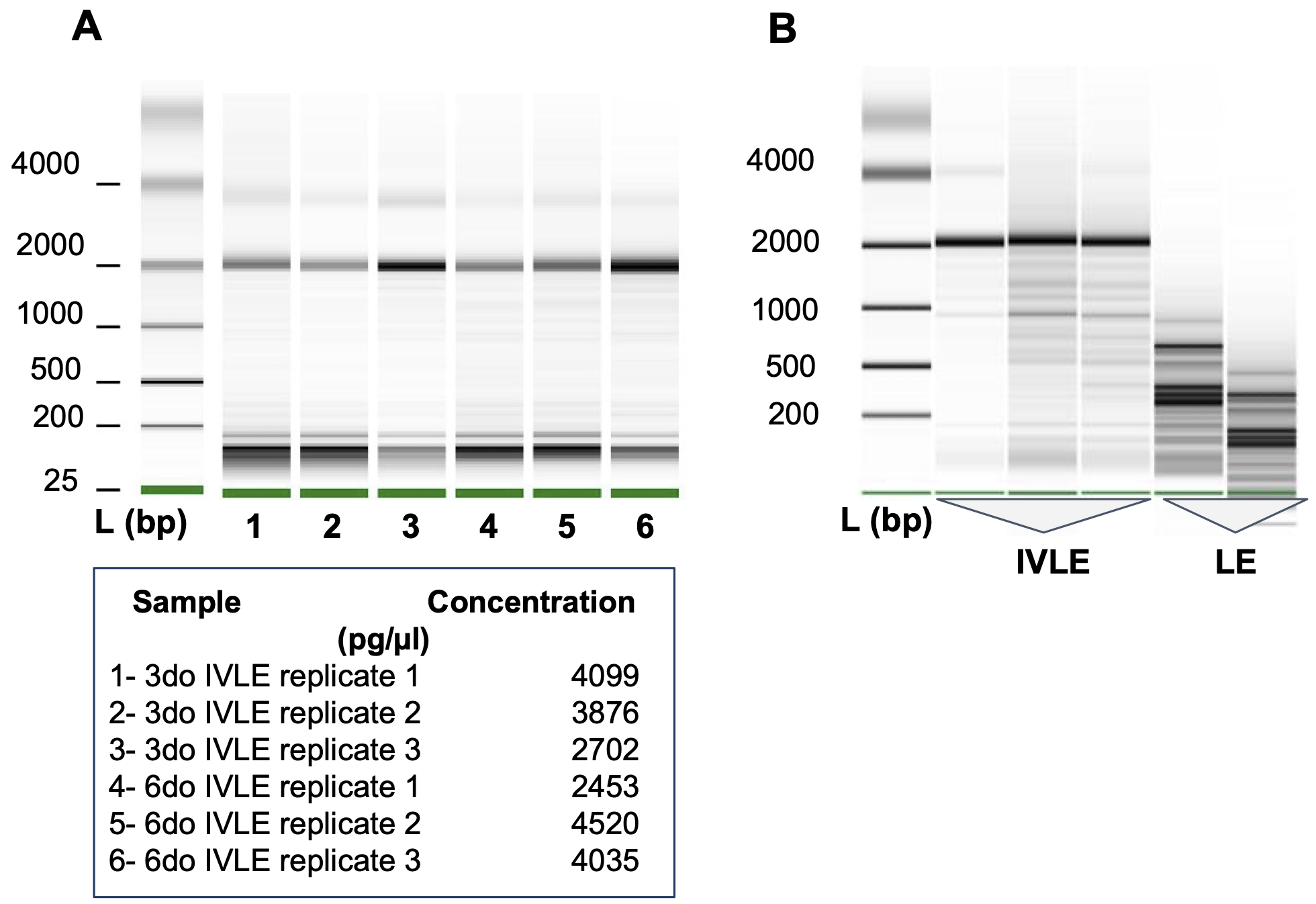

Supplement: Figure S1 — (A) Bioanalyzer electropherogram of RNA preparations isolated from D3 and D6 IVLE and processed for sequencing using the Smart-Seq2 protocol. Samples and concentrations are indicated in the bottom panel. (B) Representative bioanalyzer traces of RNA preparations isolated from IVLE and liver eggs (LE), as indicated. [file EMS156637-supplement-Figure_S1.TIFF]

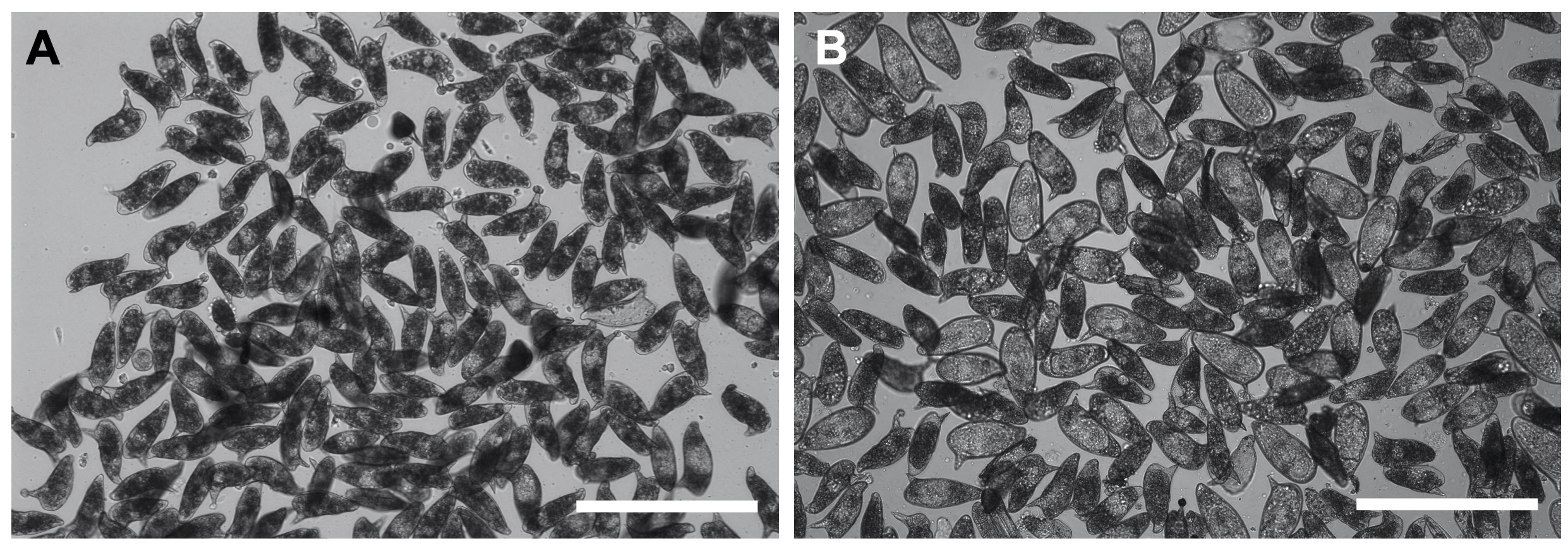

Supplement: Figure S2 — Representative micrographs of D3- (A) and D6 (B) IVLE. Scale bar: 300 μm. [file EMS156637-supplement-Figure_S2.TIFF]

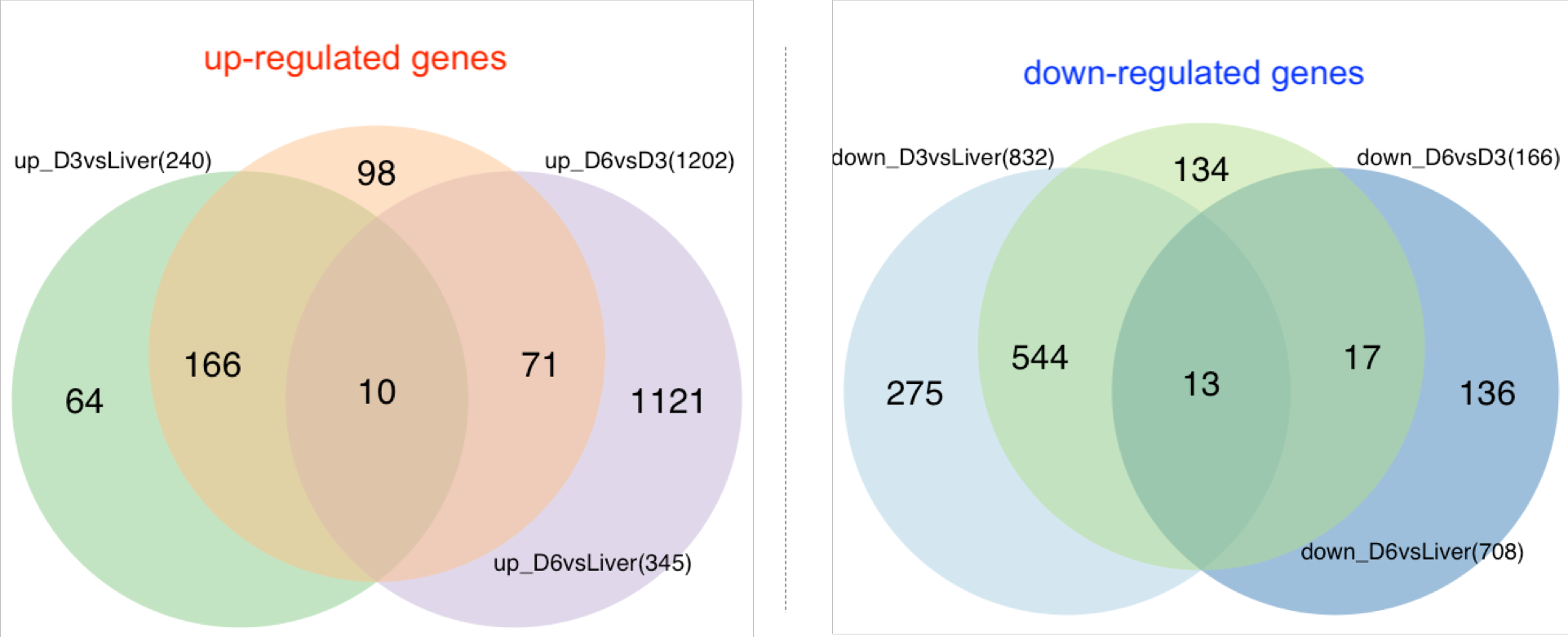

Supplement: Figure S3 — Left. Venn diagram indicating the number of shared/unshared upregulated genes amongst the three comparisons: D6 vs D3 IVLE (1202 genes); D3 IVLE vs liver eggs (240 genes); D6 IVLE vs liver eggs (345 genes). Right. Venn diagram indicating the number of shared/unshared downregulated genes amongst the three comparisons: D6 vs D3 IVLE (166 genes); D3 IVLE vs liver eggs (832 genes); D6 IVLE vs liver eggs (708 genes). The gene names and identifiers are provided in Supplementary Table S3. [file EMS156637-supplement-Figure_S3.TIFF]

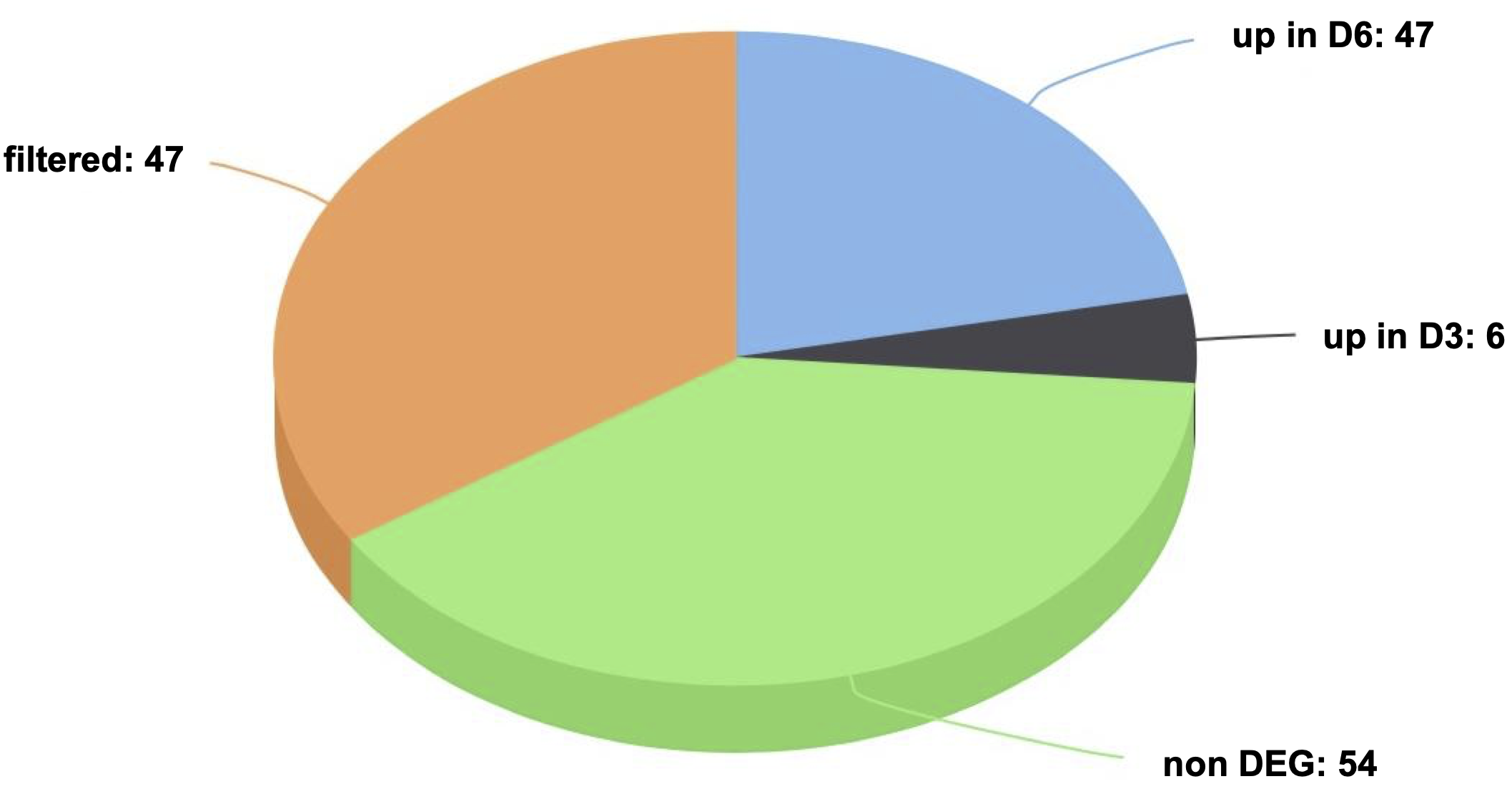

Supplement: Figure S4 — Pie chart indicating the differential expression of the top 200 miracidium-sporocyst enriched genes in D3 and D6 IVLE. Filtered: genes that were filtered out for differential expression analysis in DESeq2, as were detected as outlier genes; DEG: differentially expressed genes. [file EMS156637-supplement-Figure_S4.TIFF]
